# Human placenta induces hair regrowth in chemotherapy-induced alopecia via inhibition of apoptotic factors and proliferation of hair follicles

**DOI:** 10.1186/s12906-020-03025-z

**Published:** 2020-07-20

**Authors:** Mi Hye Kim, Kyuseok Kim, Haesu Lee, Woong Mo Yang

**Affiliations:** 1 Department of Convergence Korean Medical Science, College of Korean Medicine, Kyung Hee University, Seoul, 02447 Republic of Korea; 2 Department of Ophthalmology, Otorhinolaryngology and Dermatology of Korean Medicine, College of Korean Medicine, Kyung Hee University, Seoul, Republic of Korea

**Keywords:** Apoptosis, Chemotherapy-induced alopecia, Hair follicle, Human placenta, Proliferation

## Abstract

**Background:**

The human placenta (HP) is a complex organ used to alleviate tiredness and promote wound healing. Previous research showed the hair growth-promoting effect of HP. However, no reports have addressed the effects of HP on hair regrowth in chemotherapy-induced alopecia. In this study, we investigated the effects of HP on the apoptosis and proliferation of hair follicles in chemotherapy-induced alopecia.

**Methods:**

Male C57BL/6 mice in telogen were depilated to enter anagen. After 9 days, dystrophic catagen was induced by the intraperitoneal injection of 150 mg/kg cyclophosphamide. During 9 to 16 days, 0.1 and 1 mg/mL HP were topically applied to depilated dorsal skin.

**Results:**

Dystrophic hair follicles by cyclophosphamide were recovered by HP treatment. New hair shafts containing hair fibers appeared to be straight after HP treatment. Immunohistological staining revealed a significant increase of Ki67-positive cells in hair follicles treated with 1 mg/mL HP. Topical HP treatment increased the ratio of Bcl-2/Bax, while it attenuated the expression of pro-apoptotic Bax, p53, and cytochrome c with caspase-9 and -3. In addition, the expression of KGF and the phosphorylation of AKT were upregulated by HP treatment.

**Conclusion:**

These results suggest that HP treatment induced hair growth by inhibiting apoptosis and promoting the proliferation of hair follicles. HP may be useful for treating chemotherapy-induced alopecia.

## Background

Chemotherapy-induced alopecia (CIA) is a psychologically devastating side effect for patients common to certain therapeutic regimens in oncology [1]. Novel chemotherapies are continually being developed, but hair loss consistently ranks among their associated features that are particularly troublesome and distressing to patients. The much-feared disfigurement of massive hair loss drives 8% of patients to consider refusing chemotherapy [2]. In patients with early-stage breast cancer, almost 90% of them considers alopecia as the most burdensome aspect of perioperative chemotherapy. Some of them even regards anticipated alopecia as more difficult to cope with than the loss of a breast [3]. Thus, CIA can cause the extreme anxiety regarding body image, self-esteem and quality of life, despite its temporary nature.

Multiple therapeutic agents for treating CIA have been proposed, such as scalp hypothermia, minoxidil, and calcitriol [1]. However, scalp hypothermia is not recommended in patients with circulating malignant cells because of its potential to cause metastasis to the scalp [4]. In addition, the efficacy of minoxidil is limited and transient because hair loss resumes after its use is discontinued [5]. Finally, there are also still considerable problems associated with calcitriol, including limited supply, high manufacturing costs, excessive inflammation, and various disease risks [6]. Therefore, there is a need to develop more effective and satisfactory management strategies for CIA in the field of clinical oncology.

The human placenta (HP), a complex organ that contains enzymes, amino acids, peptides, polydeoxyribonucleotide, vitamins, trace elements and growth factors, has been used to alleviate tiredness and promote wound healing [7]. It exerts anti-oxidative, anti-inflammatory and whitening functions [8–10]. In several animal studies, the evidence was provided that HP reduced fibrosis, induced liver regeneration and regulated inflammatory responses via production of transforming growth factor-β [11]. Recently, it was also reported that HP has hair growth-promoting activity, through mechanisms acting via the Wnt/ß-catenin pathway, thereby improving hair growth in mice [5]. Despite the recent investigation of HP, its efficacy in a CIA model has not been scientifically verified. Hence, the present study was undertaken to evaluate the anti-apoptotic and hair growth-promoting effects of HP in a cyclophosphamide (CYP)-induced CIA mouse model.

## Methods

### Animals

Male C57BL/6 mice aged 7 weeks in the telogen phase with black fur were obtained from RAONBIO Inc. (Yongin, Korea). The mice were housed in community cages with 12 h light periods and fed water and mouse chow ad libitum. All experiments were approved by Committee on Care and Use of Laboratory Animals of the Kyung Hee University (KHUASP (SE)-14–028). And all procedures were performed in accordance with the regulations for the Care and Use of Laboratory Animals of the Kyung Hee University.

### Experimental design

After 1 week of adaptation, all mice were depilated with shaving cream. The mice in telogen phase judged from pink skin color were induced to enter anagen by depilation. On Day 9, 150 mg/kg CYP (Sigma, St. Louis, MO, USA) in phosphate buffered saline (PBS; Gibco, Thermo Fisher Scientific, Inc., Waltham, MA, USA) was intraperitoneally injected into all groups except normal group to induce dystrophic catagen. Mice were divided into 5 groups (*n* = 7); normal, CYP, dexamethasone (DEX), HPL (0.1 mg/mL of HP, low dose) and HPH (1 mg/mL of HP, high dose). On Day 17, hair follicles in anagen phase (black skin color) spontaneously entered into catagen (gray skin color), as can be recognized from the transformation of skin color. CYP group were topically treated vehicle (PBS) and normal group were non-treated. 0.1% DEX (Sigma), as positive control, was topically treated on Day 1, 3, 5, 7 and 9 to 16. 100 μL of HP at concentrations of 0.1 and 1 mg/mL were topically treated to depilated region on Day 9 to 16. Human placental extracts, used in this study were manufactured by UNIMED Pharm. Inc. (Asan, Republic of Korea), which has obtained Korean good manufacturing practice (KGMP) authorization (UDI 88064 9505 9927). During the experiment, body weight of all mice was normalized. In addition, mice treated with HP showed no abnormalities in the daily activity. At Day 17, all mice were sacrificed by cervical dislocation and photographed by digital camera (Sony, Tokyo, Japan).

### Histology

Dorsal skin from all mice was fixed in 10% neutralized formalin (Sigma) for 18 h. Fixed skins were dehydrated with ethanol and xylene. 4 μm cut paraffin-embedded sections were prepared with rotary microtome. Hematoxylin and eosin (H&E) solution (YD Diagnostics Corp., Yongin, Korea) was applied to the slides to monitor the hair regrowth. The slides of each group were evaluated by randomly selecting 5 photographs per mice. Digital images were obtained using the Leica Application Suite Microscope Software (Leica Microsystems Inc., IL, USA). The used magnification was × 100.

### Immunohistochemistry

For antigen unmasking, the slides were boiled in 10 mM sodium citrate buffer (Gibco). The slides were treated with 3% hydrogen peroxide (Biosesang Corp., Sungnam, Korea) for 15 min to reduce endogenous peroxidase and with normal goat serum to minimize non-specific binding. To detect the proliferative cells, slides were treated by anti-mouse ki67 IgG and biotinylated anti-rabbit antibody (Cell Signaling Technology, Danvers, MA, USA). The stained tissues by avidin/biotinylated enzyme complex kit (Vector Laboratories, Inc., Burlingame, CA, UK) were developed using the 3,3-diaminobenzidine substrate (Sigma). The slides of each group were evaluated by randomly selecting 5 photographs per mice. Digital images were obtained using the Leica Application Suite Microscope Software (Leica Microsystems Inc.). The used magnification was × 200.

### Western blot analysis

The protein lysates from dorsal skins (*n* = 7) were prepared with lysis buffer containing protease inhibitors (Roche, Hoffmann, USA). Thirty micrograms of protein were electrophoresed in a sodium dodecyl sulfate polyacrylamide gel electrophoresis and transferred onto a polyvinylidene fluoride membrane. Each primary antibodies, which were protein kinase B (PKB, also called AKT), bcl-2-associated X protein (Bax), b cell leukemia protein-2 (Bcl-2), cytochrome c (Cyt c), caspase-9 and -3 and keratinocyte growth factor (KGF) and anti-rabbit alkaline phosphatase-conjugated secondary antibodies were added to the membranes. All primary and secondary antibodies were purchased from Cell Signaling Technology. Bound antibodies were visualized using an enhanced chemiluminescence detection reagent (Amersham Pharmacia, Piscataway, NJ, USA). The protein bands were cropped to remove the unnecessary backgrounds and show clear blots (Additional file 1). The bands were quantified using a computerized densitometry system Image J (NIH, Bethesda, MD, USA).

### Statistical analysis

Significance was determined by one-way analysis of variance (ANOVA) and Dunnett’s multiple comparison tests. In all analyses, *P < 0.05* was taken to indicate statistical significance.

## Results

### HP recovers dystrophic hair follicles

**Fig. 1:**
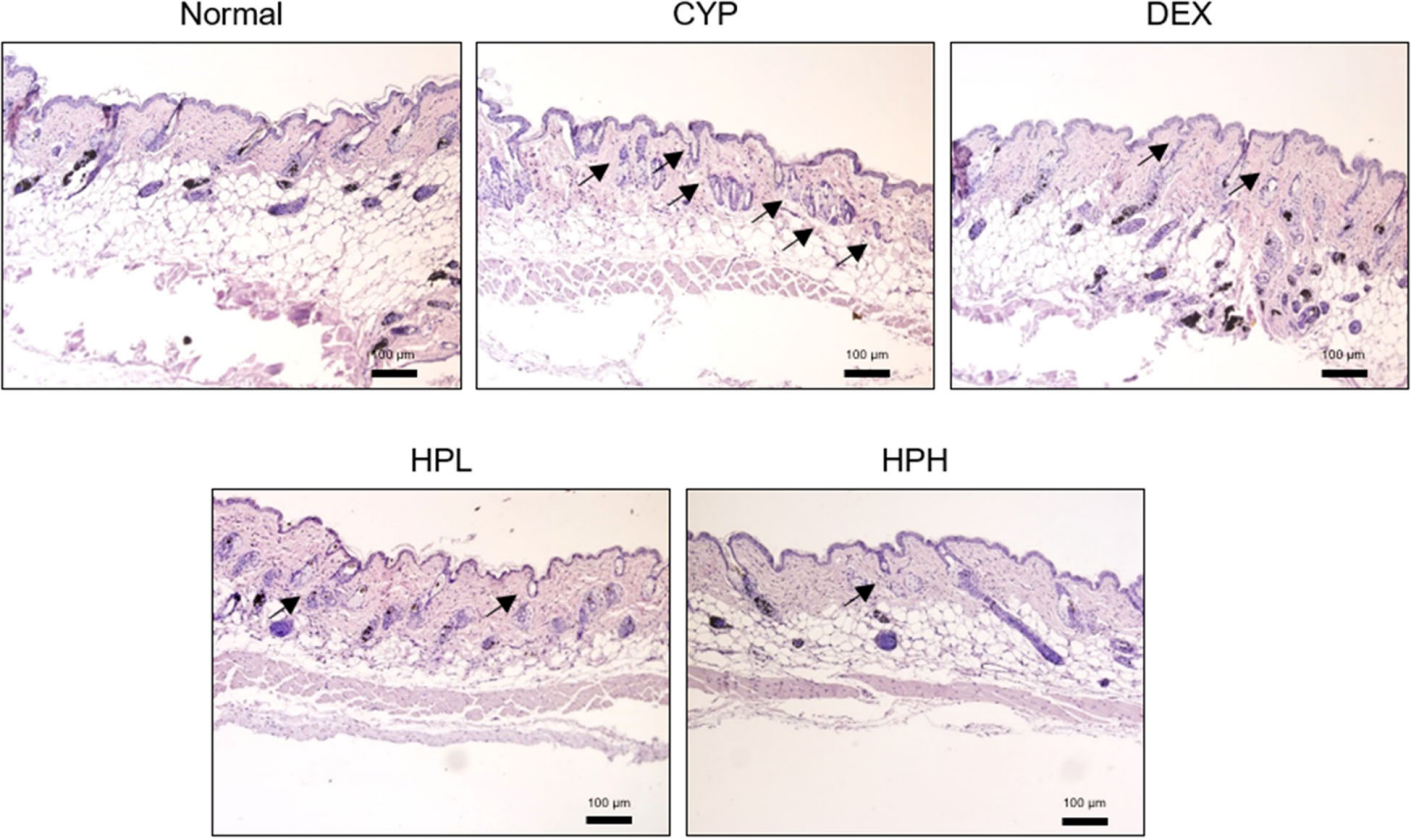
Histological changes of dorsal skins by hematoxylin and eosin staining. The used magnification is × 100. Arrows indicate the dystrophic hair follicles

Broken hair shafts did not emerge from hair follicles on the skin surface in the CYP group. In addition, CYP group showed that miniaturized hair follicles contained no hair fibers. Treatment with HP led to the recovery of tapering hair shaft length and broken hair follicles (Fig. 1). After HP treatment, new hairs on the surface of the epidermis appeared to be straight, especially in skin treated with 1 mg/mL of HP. Reversed miniaturized hair follicles showed hair fibers upon treatment with 1 mg/mL of HP.

### HP inhibits apoptosis-related factors

**Fig. 2:**
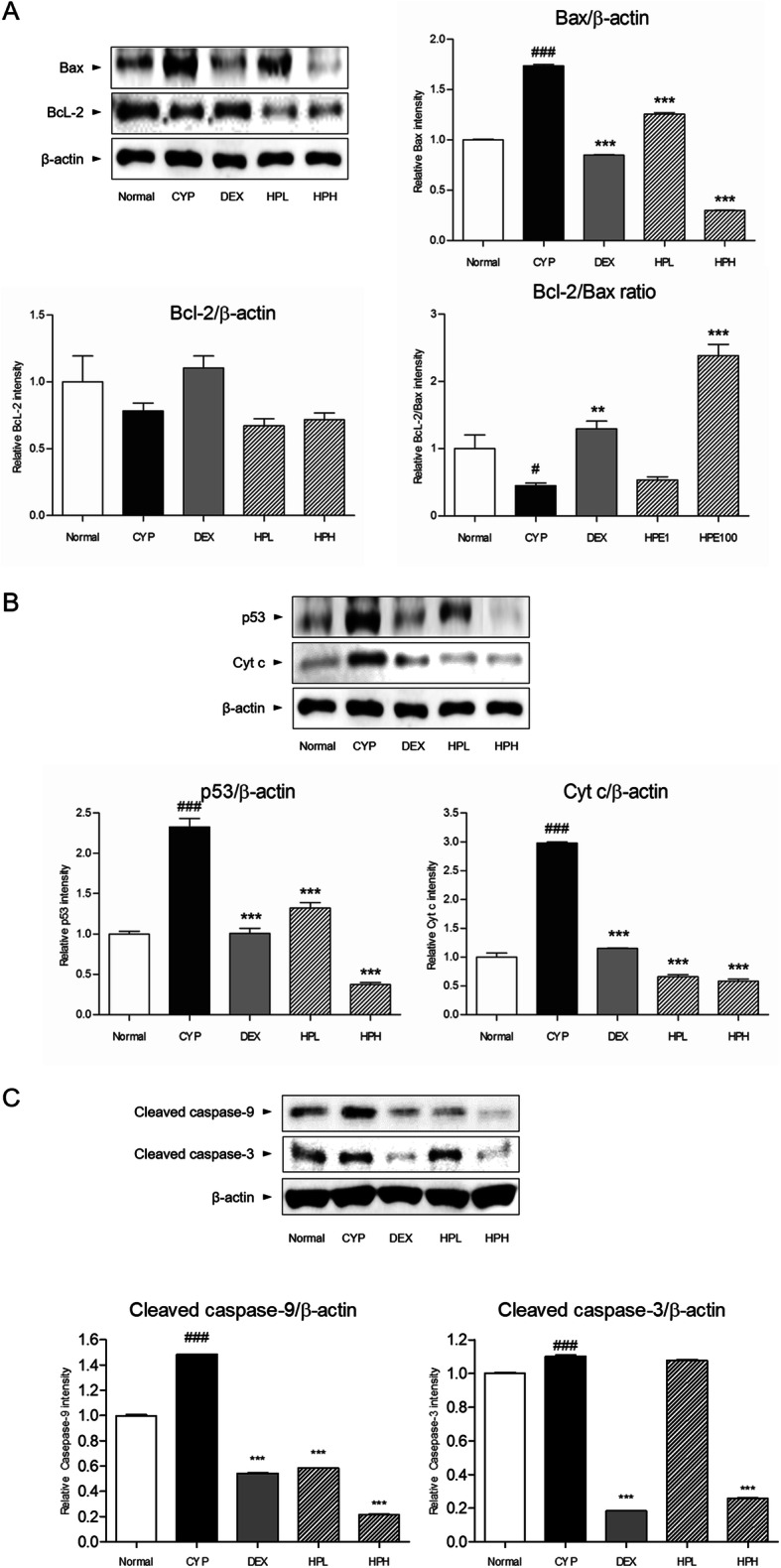
Expression of Bax and Bcl-2 (**a**), and p53 and Cyt c (**b**), and caspase-9 and -3 (c). Data shown are mean ± S.E.M. Mean values were significantly different for the following comparisons. ^###^*p* < 0.001, ^#^*p* < 0.5 versus Normal; ^***^p < 0.001, ^**^p < 0.01 versus CYP

There was a notable increase in the level of Bax in the CYP group compared with that in the normal group. Treatment with 0.1 and 1 mg/mL HP significantly decreased the expression of Bax in a dose-dependent manner. The expression of Bcl-2 was unchanged by HP treatment. The Bcl-2/Bax ratio was significantly increased in the HPH group (*p* < 0.05), while it was decreased in catagen-affected skin (Fig. 2a). In addition, the level of p53 was significantly increased in the CYP group compared with that in the normal group. After treatment with 0.1 and 1 mg/mL HP, there were significant decreases in the level of p53 in comparison with that upon CYP treatment (*p* < 0.05). Similarly, the dystrophic catagen hair follicles contained a high level of Cyt c; this was significantly ameliorated by treatment with 0.1 and 1 mg/mL HP (Fig. 2b). Moreover, the expressions of caspase-9 and caspase-3 in dorsal skins were significantly decreased by 1 mg/mL HP (*p* < 0.05) (Fig. 2c).

### HP promotes the proliferation of hair follicles

**Fig. 3:**
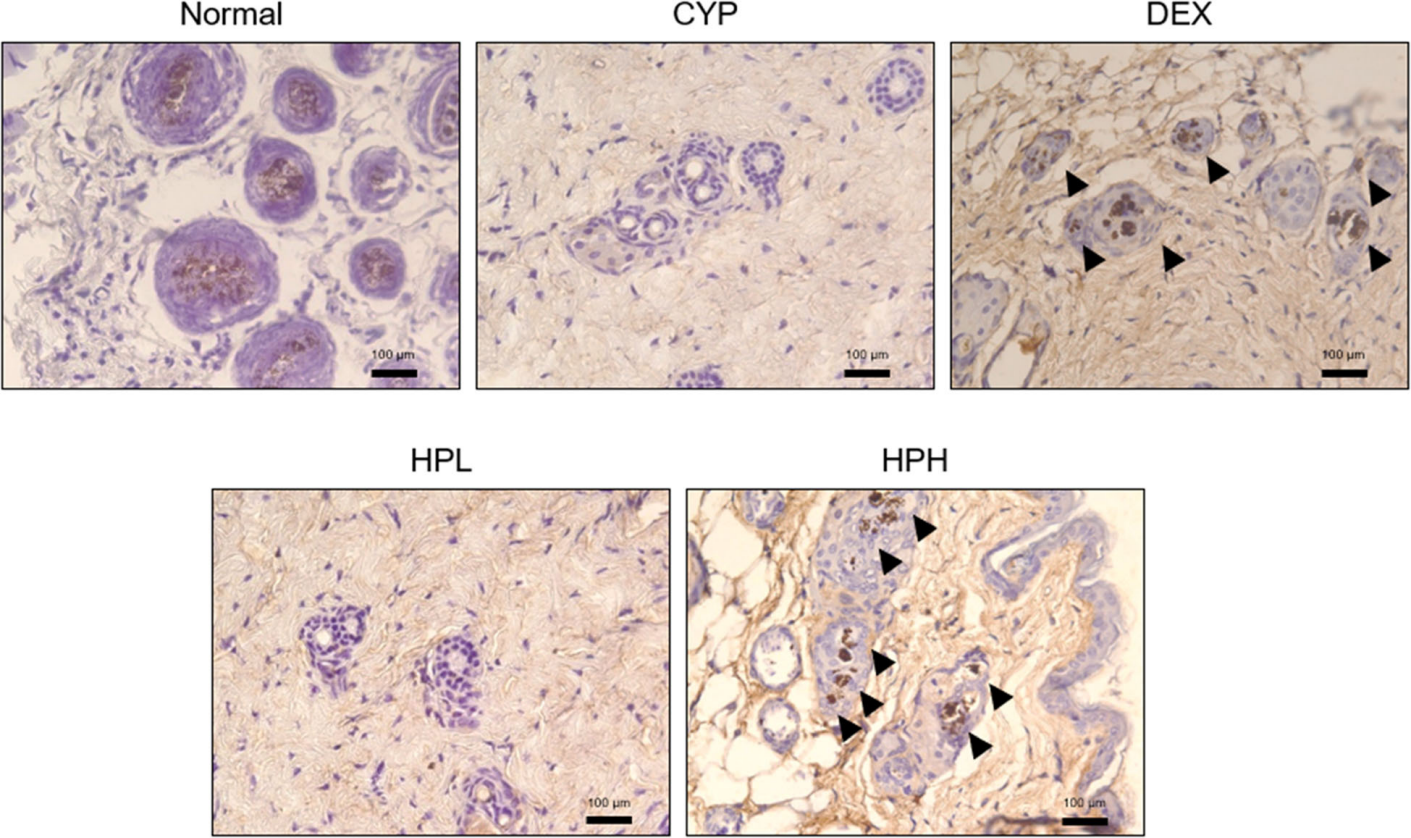
Expression of Ki67-positive cells in hair follicles. Brown stained cells by 3,3-diaminobenzidine substrate were appeared in the hair follicles matrix as well as dermal papilla. The used magnification is × 400. Arrow heads indicate the Ki67-stained cells

The follicular proliferative pattern in hair keratinocytes was determined by analyzing Ki67-positive cells. In CYP-induced dystrophic hair follicles, few Ki67-positive cells were seen in the hair follicle matrix. While there was no change of Ki67-positive cells in the HPL group, treatment with 1 mg/mL HP markedly increased the Ki67-positive proliferative cells (*p* < 0.05) compared with those in the CYP group (Fig. 3).

### HP increases KGF expression and AKT phosphorylation

**Fig. 4:**
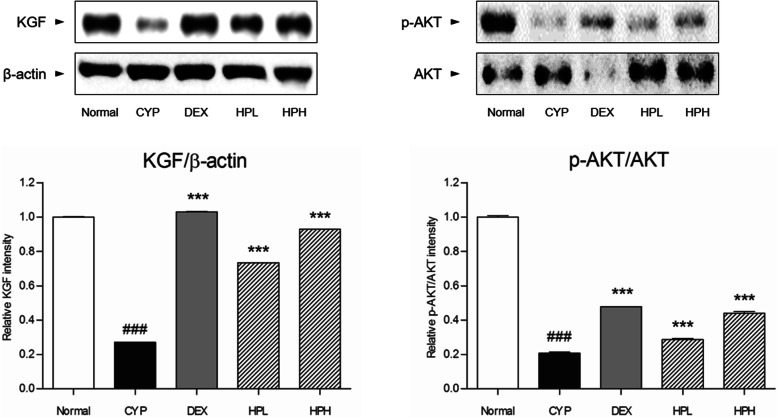
Expression of KGF and AKT. Data shown are mean ± S.E.M. Mean values were significantly different for the following comparisons. ^###^*p* < 0.001 versus Normal; ^***^p < 0.001 versus CYP

A significant decrease on the level of keratinocyte growth factor (KGF) was shown in dystrophic catagen hair follicles. The expression of KGF was dose-dependently increased by treatment with 0.1 and 1 mg/mL HP (*p* < 0.05). Following the results regarding KGF expression, the phosphorylation of protein kinase B (PKB, also called AKT) in the HPL and HPH groups was ameliorated (*p* < 0.05), while that in the CYP group was reduced (Fig. 4).

## Discussion

The hair cycle is usually divided into three phases; anagen (growth), catagen (regression) and telogen (resting) [12]. Because hair follicles affected by chemotherapy in the anagen phase immediately enter the dystrophic catagen stage, CIA is recognized as anagen effluvium [13]. In CIA, increased fragile hair counts and miniaturized hair follicles with apoptosis-related damage have frequently been identified [14]. Therefore, the inhibition of apoptosis and the promotion of hair growth are important for treating CIA [15]. In the present study, HP treatment restored the size of hair follicles containing hair fibers. Broken hair shafts, which did not emerge through the surface of the epidermis, were restored by HP treatment.

Massive apoptosis-driven involution in the dystrophic catagen phase is characterized by a decrease of the ratio of Bcl-2 to Bax. Bcl-2, an inhibitor of apoptosis, interacts with pro-apoptotic Bax [16]. While Bcl-2 is expressed in the hair matrix and involved in hair growth, Bax promotes apoptotic cell death [17]. Bax expression is markedly increased in hair matrix keratinocytes under apoptosis [18]. Accompanied by these changes, it was reported that p53 regulates the expression of Bax, resulting in promotion of the apoptotic pathway in the hair matrix [19]. The activation of Bax by p53 stimulates Cyt c in mitochondria. Finally, the release of Cyt c from mitochondria is linked to apoptosis. In addition, caspase-9 was recruited from release of Cyt c into the cytoplasm, resulting in the formation and activation of caspase-3 [18]. In this study, we found that HP treatment decreased the expression of Bax. Accordingly, the ratio of Bcl-2/Bax was significantly upregulated in HP-treated skin. Also, HP attenuated the expression of p53. Furthermore, the expressions of caspase-9 and -3 in dorsal skin were markedly declined by topical HP application. Consistent with these results, the release of Cyt c from mitochondria was restricted by HP treatment.

Apart from apoptosis, the growth of hair follicles is vital for the recovery of hair. KGF is functionally essential for hair follicle development [20]. KGF has been known to promote hair transition into the anagen stage as well as hair follicle proliferation. The activation of KGF induces activation of the AKT pathway, resulting in the proliferation of hair follicles [21]. The inhibition of AKT phosphorylation is regarded as being associated with an inability of damaged to the hair follicles to undergo proliferation [22]. Ki67, a marker of proliferation, was positively expressed in hair follicles treated with 1 mg/mL HP. To clarify the molecular mechanism behind the involvement of HP in hair growth, the expression of KGF and the phosphorylation of AKT in dorsal skin were analyzed. HP treatment caused increases of KGF expression and AKT phosphorylation, demonstrating that HP promotes hair growth with hair follicle proliferation.

## Conclusion

The obtained results indicate that HP attenuated hair follicle apoptosis as well as promoted hair follicle proliferation. At present, the main therapeutic target-pathway of HP between inhibition of apoptosis of hair follicles and proliferation of hair follicles is unclear. In addition, it is difficult to clarify which compound acts on which mechanism because natural materials such as HP contain various compounds. There is also the possibility that multiple compounds in HP act on multiple targets at the same time. Therefore, to obtain a deeper understanding of its mode of action, a follow-up study on HP is required.

In summary, HP ameliorated massive apoptosis by inhibiting pro-apoptotic Bax, p53, Cyt c and caspase and promoted the proliferation of hair follicles via increases of KGF and the phosphorylation of AKT. Taken together, HP induced hair regrowth in the CIA-affected dystrophic catagen phase via inhibition of apoptotic factors and proliferation of hair follicles. Nevertheless, hair loss might be recurred after treatment stop of HP. Further studies will be needed to elucidate the mechanism of action and confirm the efficacy of long-term treatment of HP for CIA.

## Supplementary information

**Additional file 1.** Uncropped images of blot with prestained protein markers performed by Western blot analysis. AKT, protein kinase B; Bax, Bcl-2-associated X protein; Bcl-2, B cell leukemia protein-2; Cyt c, Cytochrome c; KGF, Keratinocyte growth factor.

## Data Availability

The datasets used and/or analyzed during the current study available from the corresponding author on reasonable request.

## Abbreviations

AKT: Protein kinase B
Bax: Bcl-2-associated X protein
Bcl-2: B cell leukemia protein-2
CIA: Chemotherapy-induced alopecia
CYP: Cyclophosphamide
Cyt c: Cytochrome c
DEX: Dexamethasone
HP: Human placenta
KGF: Keratinocyte growth factor
