# Supplementary figures and images for: Human placenta induces hair regrowth in chemotherapy-induced alopecia via inhibition of apoptotic factors and proliferation of hair follicles
Source: BMC Complement Med Ther. 2020 Jul 20;20:230. doi: 10.1186/s12906-020-03025-z (PMC7372784; doi:10.1186/s12906-020-03025-z)

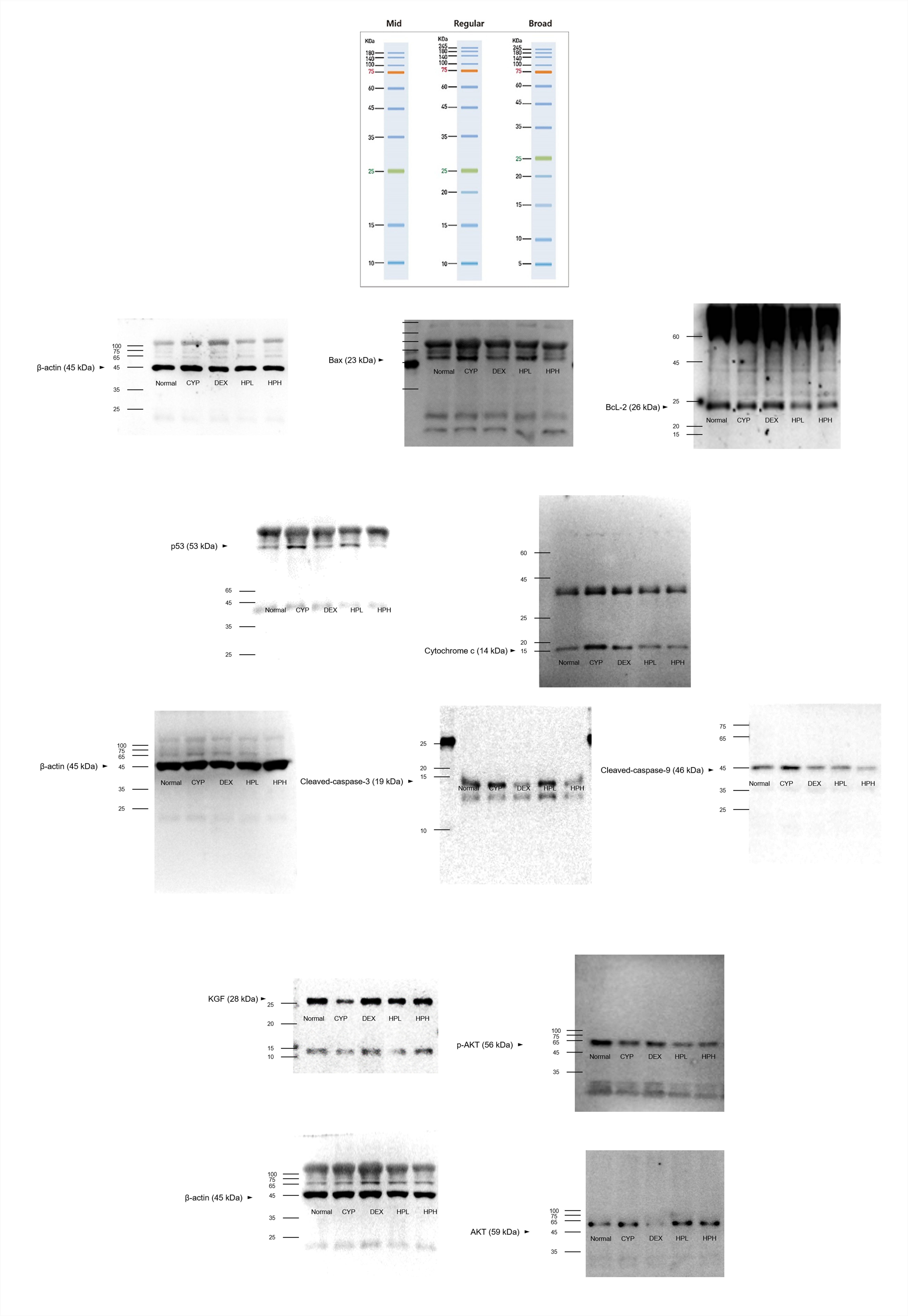

Supplement: Supplementary file 1 — Additional file 1. Uncropped images of blot with prestained protein markers performed by Western blot analysis. AKT, protein kinase B; Bax, Bcl-2-associated X protein; Bcl-2, B cell leukemia protein-2; Cyt c, Cytochrome c; KGF, Keratinocyte growth factor. [file 12906_2020_3025_MOESM1_ESM.tif]
